# The metabolomics changes in epididymal lumen fluid of CABS1 deficient male mice potentially contribute to sperm deformity

**DOI:** 10.3389/fendo.2024.1432612

**Published:** 2024-08-21

**Authors:** Xiuling Zhao, Junyu Nie, Wenwen Zhou, Xuhui Zeng, Xiaoli Sun

**Affiliations:** ^1^ Institute of Reproductive Medicine, Medical School, Nantong University, Nantong, Jiangsu, China; ^2^ Center of Reproductive Medicine, Department of Obstetrics and Gynecology, Affiliated Hospital of Nantong University, Nantong, Jiangsu, China

**Keywords:** CABS1, epididymal lumen fluid, metabolomics, sperm maturation, LC-MS

## Abstract

**Introduction:**

Epididymal lumen fluids provides a stable microenvironment for sperm maturation. Ca^2+^ binding protein CABS1 is known to maintain structural integrity of mouse sperm flagella during epididymal transit of sperm. Besides, CABS1 was reported to contain anti-inflammatory peptide sequences and be present in both human saliva and plasma. However, little is known about the role of CABS1 in regulation of the microenvironment of epididymal lumen fluids.

**Methods:**

To further confirm the role of CABS1 in epididymis, we identified the expression of CABS1 in epididymal lumen fluids. Moreover, high performance liquid chromatography, coupled with tandem mass spectrometry technique was used to analyze the metabolic profiles and *in vivo* microperfusion of the cauda epididymis and inductively coupled plasma mass spectrometry (ICP-MS) assays was used to detect the concentration of metal ion of mouse cauda epididymal lumen fluids in CABS1 deficient and normal mice.

**Results:**

The results showed that CABS1 is present in epididymal lumen fluids, and the concentration of calcium in epididymal lumen fluids is not changed in *Cabs1^-/-^* male mice. Among 34 differential metabolites identified in cauda epididymis, 21 were significantly upregulated while 13 were significantly downregulated in KO cauda epididymis. Pathway analysis identified pyrimidine metabolism, inositol phosphate metabolism, arachidonic acid metabolism, purine metabolism and histidine metabolism as relevant pathways in cauda epididymis.

**Discussion:**

The perturbations of mitochondrial dysfunction and inflammation may be the crucial reason for the poor performance of *Cabs1^-/-^* sperm.

## 1 Introduction

Testicular spermatozoa are morphologically complete when released, but largely immature and not yet capable of motility and sperm-egg recognition (1). During sperm transit in the epididymis, a series of changes are also taking place in different segment, such as the migration of cytoplasmic droplets (2), further changes in the morphology of sperm (3), changes in the lipid composition of sperm membranes (4). Sperm undergo a series of physicochemical and morphological changes from testis to epididymis, leading to the final attainment of full maturation.

The epididymis is divided into four main anatomical segments: the initial segment, caput, corpus, and cauda, which are specialized for its unique characteristics and functions and provides an optimal intraluminal microenvironment for sperm maturation. The luminal fluid consists of multiple components including proteins, RNA, inorganic ion, and small organic molecules, many proteins have been demonstrated to play significant role in sperm maturation (5). The precise regulation of secreted proteins which are in direct contact with the spermatozoa in the lumen of the epididymis is essential for the sperm maturation process. It has been found that over 700 proteins are added and more than 1000 proteins are removed from sperm during the transit from caput to the cauda in epididymis (6, 7). Abnormal epididymal function may account for a significant proportion of idiopathic male infertility, particularly in infertile patients with normal spermatogenic processes in the testes. The progress of metabolomics techniques has helped identify more metabolites, which yield a better understanding of the physiological processes at metabolite levels. In recent studies, over thousands of different metabolites were identified in different segment of epididymis lumen fluid using quantitative and qualitative comparisons.

The particular ionic composition in epididymal fluids is closely related to the regulation of luminal environment that helps sperm to maintain the quiescence state (8, 9). A decrease gradient of luminal Ca^2+^ was observed along the epididymal duct (10–12), the low calcium levels are believed to be essential to help keep the sperm in the dormant stage (13, 14). The aberrant epididymal luminal calcium homeostasis can lead to defective spermatozoa and impaired fertility (14–16). As a calcium-binding protein, whether CABS1 is involved in the regulation of Ca^2+^ homeostatic regulation or other components in the lumen is not clear.

The calcium-binding protein spermatid-associated 1 (CABS1) was found to be enriched in mammalian, mainly in elongated spermatid of testes and maturation sperm of epididymis in mice (17, 18). Moreover, our previous study showed that CABS1 had no effect on spermatogenesis in testis, while CABS1 was required for the maintenance of structural integrity of sperm flagella during epididymal transit (18). The increased percentage of sperm with a bent tail in the epididymis of *Cabs1* deficient mice suggested that the epididymal lumen fluid may account for the sperm tail deformation during the normal transit of spermatozoa in the epididymis. In an attempt to fully illuminate the role of CABS1 in regulation sperm maturation in epididymis, we employed *in vivo* microperfusion of the cauda epididymis and inductively coupled plasma mass spectrometry (ICP-MS) assays and untargeted liquid chromatography-mass spectrometry (LC/MS) analysis on wild type and CABS1 deletion mice to reveal the changes in the epididymis microenvironment.

## 2 Materials and methods

### 2.1 Animals

The *Cabs1* KO C57/BL6 mice were generated as is shown in Zhang et al. (18), and WT mice were bred by ourselves. Healthy 4~5-month-old male C57/BL6 mice were sacrificed for collecting the epididymal luminal fluid. The experiment was conducted in accordance with the guidelines approved by the Institutional Animal Ethical Committee (IAEC) of Nantong University (S20230101-005, Nantong, China).

### 2.2 Epididymal fluid collection

WT (n=6) and KO (n=6) male mice were sacrificed, and the cauda epididymis was removed and immediately kept in 200 μL DPBS, several cracks were made in the tubules to facilitate the release of the epididymal fluid into the DPBS. After an incubation period at 37°C for 15 min, the DPBS containing the epididymal fluid and sperm was collected and centrifuged at 1500 rpm for 5 min and 12000 rpm for 10 min at 4°C. The resulting supernatants were collected and stored at -80°C for LC-MS analysis after fast freezing using liquid nitrogen.

### 2.3 Sample preparation

Samples were thawed on ice. All samples were concentrated in lyophilizer, and 100 μL of 80% methanol was added to the concentrated precipitate. The mixtures were vortexed for 1 min, sonicated for 30 min at 4°C, and placed at -20°C for 1 hour. Afterward, the mixtures were centrifuged at 12000 rpm for 15 min. A total of 200 μL of the supernatant was transferred to a 1.5 mL tube along with 5 μL 2-chloro-l-phenylalanine (1 mg/mL). Finally, the mixtures were transferred to a glass vial.

### 2.4 LC-MS analysis

LC-MS analysis was performed on a 15 μL aliquot of the pretreated epididymal cauda fluid samples using a LC-Q/TOF-MS platform (1290 Infinity LC, 6530 UHD and Accurate-Mass Q-TOF/MS; Agilent, Santa Clara, CA, USA) with the column (2.1 mm × 100 mm × 1.8 μm; Agilent, Santa Clara, CA, US) held at 40°C. The mobile phase consisted of A (water with 0.05% formic acid) and B (acetonitrile). The flow rate was set to 0.4 mL/min, and the column temperature was maintained at 40°C. The gradient elution program was as follows: 5% B for 0 to 1 min, 95% B for 1 to 13.5 min, and then stable at 5% B for 13.6 to 16 min.

To identify differential metabolites in the epididymal cauda fluid of WT and KO mice, both positive and negative modes of electrospray ionization source (ESI) were employed. The ionization mode was set with a capillary voltage of 3.0 kV (+) and 3.2 kV (-), a heater temperature of 300°C, sheath gas flow of 45 arb, auxiliary gas flow of 15 arb, sweep gas flow of 1 arb, a capillary temperature of 350°C, and an S-Lens RF level of 30% (+) or 60% (-).

### 2.5 Bioinformatics and statistical analysis

The data were analyzed using SIMCA-P 14.1 software package (Sartorious Stedim Data Analytics AB, Umea, Sweden). Principal component analysis (PCA) and orthogonal partial least-squares discriminant analysis (OPLS-DA) were performed on the data. The first principal component of the variable importance in the projection (VIP) was utilized to identify differential metabolites, where the VIP value exceeded 1 and the p-value of the Student’s t-test was <0.05. Additionally, the Kyoto Encyclopedia of Genes and Genomes (KEGG) database (http://www.genome.jp/kegg/) was used to identify the enriched metabolic pathways of differential metabolites.

### 2.6 *In vivo* microperfusion of the cauda epididymis and inductively coupled plasma mass spectrometry assays

To collect the epididymis lumen fluid and prevent the contamination of calcium ions in the blood, we employed *in vivo* microperfusion, following established methods (16). Briefly, adult male mice were anaesthetized via intraperitoneal injection of sodium pentobarbital. The distal epididymis was exposed through an abdominal incision under a dissecting microscope, and the vas deferens was cannulated with a microtubule. Another microtubule was inserted into the lumen of the cauda tubule, and paraffin oil was infused at a rate of approximately 100 microliters per minute using an infusion pump (Genie Touch, Kent Scientific). The collected sperm-fluid samples were initially weighed and then diluted 10 times (w/v) with 10 mM Tris-HCL (pH 7.4), followed by centrifugation at 3000 x g. The resulting supernatant was transferred to a new eppendorf tube. An equal volume of nitric acid (69%, Damas-beta) to the supernatant and sperm pellet, respectively, was added for digestion. Samples were boiled for 15 min at 100°C and then stored for ICP-MS at 4°C. A quadrupole-based NexION 2000 S ICP mass spectrometer equipped with a TYPE C ST3 nebuliser and a quartz cyclone spray chamber (PerkinElmer) was utilized throughout the experiment. Prior to analysis, the ICP-MS underwent adjustment using a multi-element standard solution of 10 mg/L. Samples were diluted with deionized water and measured in dynamic reaction cell (DRC) mode using NH3 as the reaction gas to eliminate interferences. 1 μg/L of Ge was used as an internal standard.

### 2.7 ATP content in epididymal sperm

Sperm ATP content was measured using an enhanced ATP assay kit, S0027 (Beyotime Biotechnology, Shanghai, China). To accomplish this, 200 μL of cell lysis reagent was added to the sperm collected from one mouse cauda. The sperm suspension was subsequently vortexed and then incubated at room temperature for 5 min. The resulting cell lysate was centrifuged at 12000 g for 5 min, and the supernatant was utilized for ATP quantification following the manufacturer’s instructions. The concentration of ATP was calculated based on an ATP standard curve and expressed as nmol/10 μg protein.

### 2.8 Western blot

After induction of anesthesia, the epididymis was removed and different segment of epididymides were immediately kept in 100 μL DPBS, several cracks were made in the tubules to facilitate the release of the epididymal fluid. After an incubation period at 37°C for 15 min, the DPBS containing the epididymal fluid and sperm was collected and centrifuged at 1500 rpm for 5 min and 12000 rpm for 10 min at 4°C. The resulting supernatants were collected. The protein present in epididymal luminal fluid was precipitated using acetone and then centrifuged at 12000 rpm for 30 min at 4°C, the resulting supernatants were discarded, and the precipitate was dissolved in radio immunoprecipitation assay (RIPA) buffer (Beyotime Biotechnology, Shanghai, China), which contained 2% SDS. This step was performed along with the epididymal fluid to isolate sperm proteins. The proteins were separated on SDS-PAGE gels and transferred to polyvinylidene fluoride (PVDF) membranes. To block the membranes, 5% non-fat milk was used for 2 hours at room temperature. Primary and secondary antibodies were used for probing the membranes. The primary antibodies included rabbit anti-CABS1(produced by ourselves) at a dilution of 1:500, α-Tubulin (#11224-1-AP, Proteintech, Wuhan, China) and GAPDH (#10494-1-AP, Proteintech, Wuhan, China) at a dilution of 1:5000. And the secondary antibodies were diluted into 1:10000. The protein bands were visualized using Amersham Imager 600 (GE Healthcare).

### 2.9 Statistical analysis

Results are expressed as the mean ± standard error of the mean(SEM). All statistical analyses were performed using GraphPad Prism (version 7.0), and sets of data were compared using a one-tailed Student’s t-test. *P<0.05 was regarded as statistically significant, and **P<0.01 was considered extremely statistically significant.

## 3 Results

### 3.1 Genetic deletion of *Cabs1* doesn’t impact elemental contents in luminal fluid

**Figure 1:**
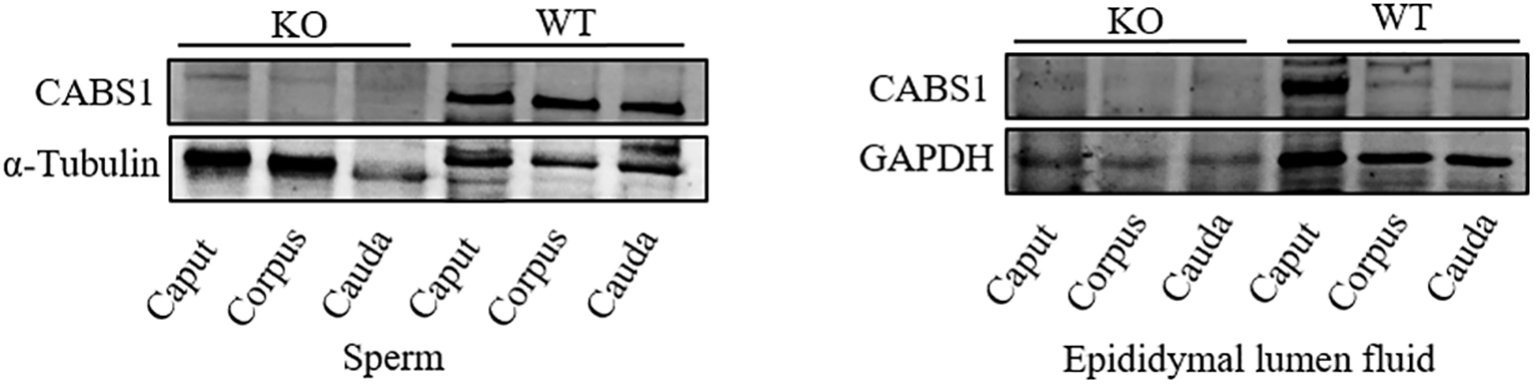
The expression levels of CABS1 in the epididymis. Immunoblot of CABS1 proteins in sperm and lumen fluid of the epididymis from WT and KO mice. The bands of expected size of CABS1 (∼68 kDa), α-Tubulin (∼55 kDa) and GAPDH (∼36 kDa). α-Tubulin and GAPDH serves as the loading control.

**Figure 2:**
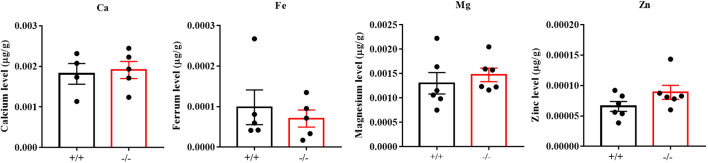
The contents of the elemental (calcium Ca, magnesium Mg, zinc Zn, and iron Fe) in the luminal fluid separated from the luminally perfused cauda-vas deference tubules of KO or WT mice (n=4 to 6 biological replicates). Amongst the detected elements, the content of Ca, Mg, Zn, and Fe showed no significant differences in the cauda luminal (*P*>0.05).

To verify the function of CABS1 in the epididymis lumen, we assessed the presence of CABS1 and the elemental composition in the luminal fluid. Western blot analysis confirmed the abundant presence of CABS1 in the epididymal luminal fluid (**Figure 1**). The elemental contents (calcium Ca, magnesium Mg, zinc Zn, and iron Fe) in the luminal fluid from the luminal perfused cauda-vas deference tubules of KO or WT mice were detected using ICP-MS. Among the commonly detected elements, no significant differences were observed in the elemental content of the luminal fluid, including calcium in the cauda (unpaired t-test, ns, *P*>0.05) (**Figure 2**).

### 3.2 PCA and OPLS-DA analysis of metabolites identified in caudal epididymal luminal fluid

**Figure 3:**
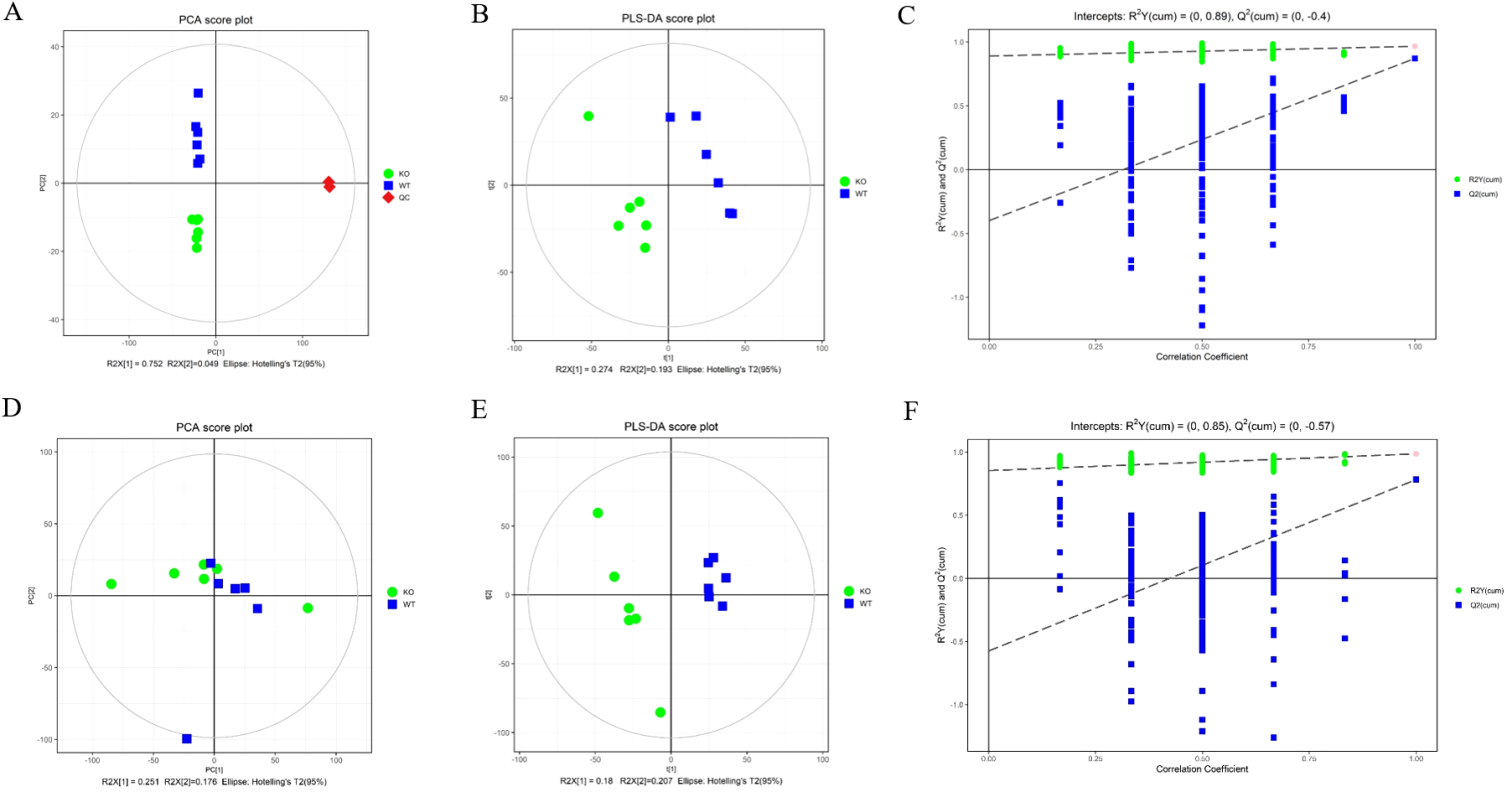
Principal component analysis (PCA) score plots **(A, D)**, orthogonal projections to latent structures-discriminate analysis (OPLS-DA) score plots **(B, E)**, and permutation tests of OPLS-DA mode **(C, F)** of epididymal fluid metabolomics analysis. **(A–C)** ESI+ model. **(D–F)** ESI- model.

To investigate the role of CABS1 in regulating the micro-environmental homeostasis of the epididymis, we compared the metabolites between WT and KO mice. The similarity and differences among the WT and KO mice were analyzed using multivariate analysis, primarily including the PCA and OPLS-DA, which allowed us to assess the stability of the overall analytical process and distribution between samples. The PCA score plots clearly distinguished the samples from different groups, with samples within the same group forming closer clusters, and the two groups being well separated (**Figures 3A, D**). To minimize intra-group errors and random errors, an OPLS-DA supervised model was utilized to discern inter-group differences and identify differential compounds. The OPLS-DA score plots demonstrated strong clustering of the two groups, with all samples falling within the 95% confidence interval (Hotelling’s T-squared ellipse) (**Figures 3B, E**). The R2 and Q2 values from the permutation test indicated that the OPLS-DA model exhibited good repeatability and predictive capabilities (**Figures 3C, F**).

### 3.3 Screening and identification of differential metabolites

Multivariate statistical analysis was conducted to identify differential metabolites associated with sperm deformity. This analysis employed the Student t-test to compare metabolite profiles in the epididymal luminal fluid between WT and KO mice. The results of this analysis can provide valuable insights into the metabolic pathways involved in sperm deformity. Based on the screening criteria, including VIP values > 1 from OPLS-DA and P-values < 0.05 from the t-test, we identified 34 differential metabolites between WT and KO mice. Among these, 21 metabolites were up-regulated and 13 were down-regulated in the epididymal luminal fluid of the cauda in *Cabs1* KO mice. The differential metabolites included fatty acids, amino acids, nucleosides, cholic acids, phospholipids, and other metabolites (**Table 1**).

**Table 1:** Differentiating metabolites between WT and KO mice in epididymal lumen fluids.

Mode	No.	Metabolites	Fold change	VIP	P-VALUE
PGs					
ESI-	1	Prostaglandin J2	2.39	1.989799	0.011599
ESI-	2	Prostaglandin D2	2.39	1.988324	0.006764
ESI-	3	15-Deoxy-Δ12,14-prostaglandin D2	2.31	1.979999	0.006856
ESI-	4	Prostaglandin E2	2.69	1.953277	0.011912
ESI-	5	13,14-Dihydro-15-keto-PGE2	1.91	1.762489	0.004588
ESI-	6	ent-Prostaglandin F2α	2.47	1.747608	0.028156
Acylcarnitines					
ESI+	7	Hexanoylcarnitine	1.68	1.5969	0.008472
ESI+	8	Acetyl-L-carnitine	1.46	1.404584	0.047536
ESI+	9	L-Carnitine	1.45	1.388692	0.023368
ESI+	10	Propionylcarnitine	1.53	1.640512	0.004706
Nucleosides					
ESI+	11	Hypoxanthine	1.37	1.555766	0.021995
ESI-	12	Thymidine	1.57	1.832369	0.018468
ESI-	13	Uridine	1.29	1.622794	0.021227
ESI-	14	Guanosine-5'-monophosphate	2.56	1.767177	0.041752
ESI+/-	15	Inosine	2.34	1.877633	0.016096
Amino acids and dipeptide					
ESI+	16	L-Methionine sulfoxide	0.46	1.97596	0.002997
ESI+	17	Gly-Tyr	1.1	1.723937	0.005169
ESI-	18	gamma-Glutamylleucine	0.66	1.486205	0.03755
ESI-	19	Leucylproline	0.68	2.032455	0.028172
ESI-	20	D-Glutamine	0.67	1.973361	0.025406
organic acid					
ESI-	21	13-HPODE	0.45	2.313781	0.000442
ESI-	22	Tetradecanedioic acid	0.55	2.045986	0.027065
ESI-	23	3-Methylglutaric acid	0.58	2.032535	0.00371
ESI-	24	Hexadecanedioic acid	0.68	1.668895	0.04492
ESI-	25	Azelaic acid	0.51	1.659492	0.046438
ESI-	26	CITRAMALATE	1.32	1.565828	0.025785
ESI-	27	Orotic acid	1.48	1.718916	0.036149
ESI+/-	28	Suberic acid	0.5	2.069452	0.019755
others					
ESI+	29	Nicotinamide	1.51	1.478073	0.017481
ESI+	30	Histamine	0.6	1.762852	0.002594
ESI+	31	N-Methylhistamine	0.51	2.107697	0.000019
ESI-	32	D-Glucose 6-phosphate	1.45	1.813342	0.029537
ESI-	33	Dihydroxyacetone phosphate	1.6	1.779492	0.007019
ESI-	34	L-Kynurenine	0.74	1.760346	0.019652

### 3.4 Pathway enrichment and metabolic pathway analysis of the potential metabolic mechanism

**Figure 4:**
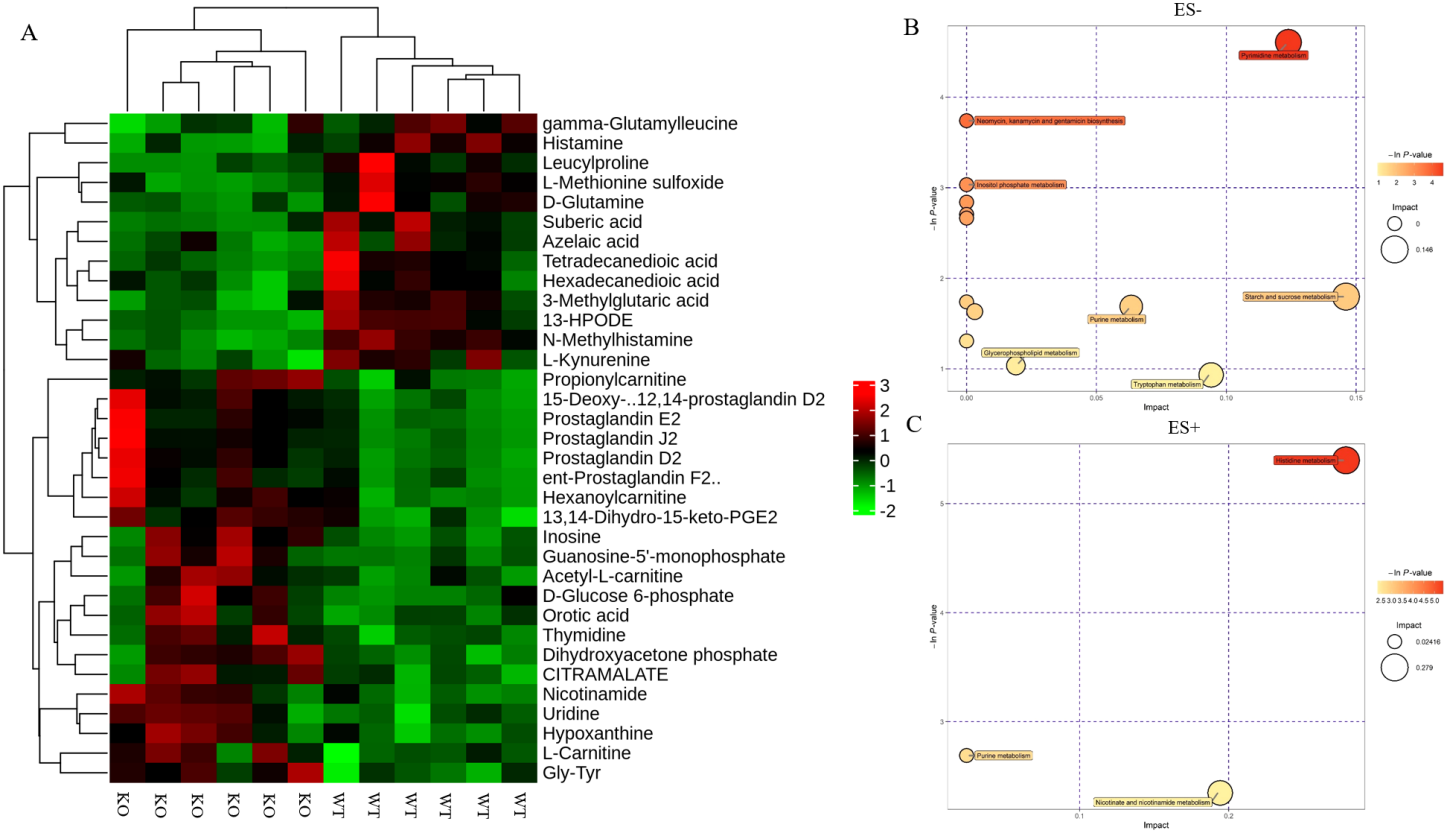
Identification of the differential metabolites in WT and KO mice. **(A)** Hierarchical cluster analysis heat-maps of identified metabolites with significant disparate levels between WT and KO mice. The relative abundance of each metabolite in each individual is depicted. Kyoto Encyclopedia of Genes and Genomes (KEGG) pathway analysis of the differentially expressed compounds for WT vs. KO (n=6). **(B, C)** Bubble plots in ES- and ES + showing the enriched metabolic pathways of varied metabolic compounds between groups, respectively. The color and Y-axis of dots are based on the -lnP-value, and the enrichment degree is more significant when the color is darker. The size and X-axis of dots represent the impact factor of the pathway in the analysis.

Following the identification of differential metabolites between WT and KO mice, we conducted an analysis of the enriched metabolic pathways using the KEGG database. The heatmap used to visualize the affected metabolites is displayed in **Figure 4A**. The pathway analysis revealed that these differential metabolites were enriched in 15 metabolic pathways (**Table 2**). **Figures 4B, C** shows that the differential metabolites were primarily involved in histidine metabolism, nicotinate and nicotinamide metabolism, starch and sucrose metabolism, pyrimidine metabolism, and tryptophan metabolism. The matching status, P-value, -ln(P), and impact of each pathway are listed in **Table 2**.

**Table 2:** Results of pathway enrichment analysis of significant metabolites.

No	Pathway	Total	Hits	Raw p	-ln(p)	Impact
1	Pyrimidine metabolism	39	3	0.010015	4.6037	0.12389
2	Neomycin, kanamycin and gentamicin biosynthesis	2	1	0.023769	3.7394	0
3	Inositol phosphate metabolism	30	2	0.048172	3.033	0
4	Linoleic acid metabolism	5	1	0.058425	2.84	0
5	Arachidonic acid metabolism	36	2	0.066888	2.7047	0
6	D-Glutamine and D-glutamate metabolism	6	1	0.069717	2.6633	0
7	Starch and sucrose metabolism	15	1	0.16574	1.7973	0.14607
8	Glycerolipid metabolism	16	1	0.17581	1.7383	0
9	Purine metabolism	66	2	0.18485	1.6882	0.06342
10	Fructose and mannose metabolism	18	1	0.19561	1.6316	0.00311
11	Glycolysis/Gluconeogenesis	26	1	0.27041	1.3078	0
12	Glycerophospholipid metabolism	36	1	0.35468	1.0365	0.01896
13	Tryptophan metabolism	41	1	0.39329	0.93322	0.09417
14	Histidine metabolism	16	2	0.0045337	5.3962	0.27868
15	Purine metabolism	66	2	0.067836	2.6907	0.02416
16	Nicotinate and nicotinamide metabolism	15	1	0.095527	2.3483	0.1943

### 3.5 Deletion of CABS1 causes abnormal level of prostaglandins and acylcarnitines

**Figure 5:**
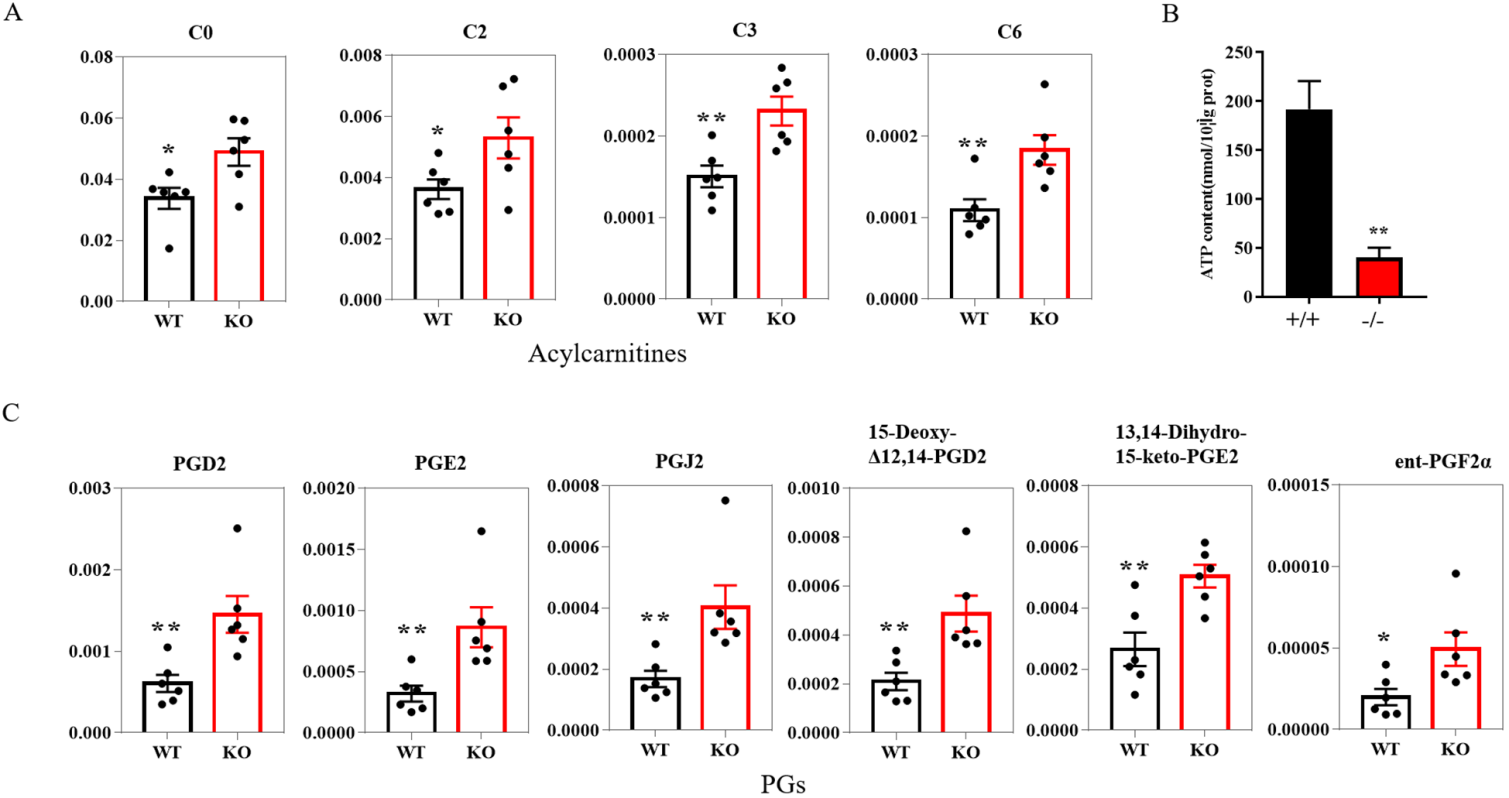
Histogram plot of the relevant metabolite in the epididymal lumen fluid of the WT and KO mice. **(A)** Acylcarnitines levels in WT and KO mice (n=6). **(B)** ATP levels in the sperm of WT and KO mice (n=3). **(C)** PGs levels in WT and KO mice (n=6). *P*-values were obtained using the T-test. *: significant *P*-values < 0.05, **: significant *P*-values < 0.01. L-carnitine (C0); Acetyl-L-carnitine (C2); Propionylcarnitine (C3); Hexanoylcarnitine (C6); PGs, prostaglandins.

Among the differential metabolites, the levels of free L-carnitine and acylcarnitines, such as hexanoylcarnitine, acetyl-L-carnitine and propionylcarnitine were all significantly increased in KO mice (**Figure 5A**). L-carnitine and acylcarnitines, known to be involved in β-oxidation, provide ATP for sperm motility. To further substantiate these findings, we measured ATP levels in sperm. Consistent with the observed metabolic alterations, our results revealed a significant decrease in ATP levels in sperm from KO mice (**Figure 5B**). Moreover, Prostaglandins and their metabolite, which are known as important inflammatory factors, such as PGD2, PGE2, PGJ2, 15-Deoxy-Δ12,14-prostaglandin D2, ent-Prostaglandin F2α and 13,14-Dihydro-15-keto-PGE2, were significantly increased in KO mice (**Figure 5C**).

## 4 Discussion

In our study, we identified the presence of the sperm-specific protein CABS1 in the epididymal luminal fluid. Additionally, our findings indicate that the deletion of CABS1 does not affect the elemental contents. However, it does have a significant impact on the levels of prostaglandins and acylcarnitines in the epididymal luminal fluid. Given acylcarnitines’ crucial role in lipid metabolism, we also quantified the ATP in sperm, our results showed that decreased ATP levels in CABS1 knockout mice may offer an explanation for their decreased motility, adding another layer of understanding to the complex factors that regulate sperm function and male fertility.

During sperm maturation in the epididymis, the dynamics of the luminal fluid components play a vital role. Interestingly, in mice with the absence of CABS1, the sperm morphology in the testes remains normal. However, abnormalities in sperm flagellar structure occur during their transit through the epididymis, and notably, this defect is independent of changes in epididymal osmotic pressure (18). Given the presence of CABS1 in the epididymal luminal fluid, further investigations were conducted to explore the potential relationship between the abnormal sperm flagellar structure in CABS1-deficient mice and alterations in the epididymal microenvironment. Analysis of the metal elements and metabolites in the epididymal luminal fluid revealed no significant changes in the levels of calcium, iron, magnesium, or zinc after CABS1 deletion (**Figure 2**). Furthermore, CABS1 contains anti-inflammatory peptide sequences and is detected in both human saliva and plasma (19, 20). However, the lack of CABS1 may indicate an inflammatory state in the microenvironment of epididymal luminal fluids, which significantly contributes to reduced sperm motility. Therefore, we conducted a metabolomic analysis to identify changes in the epididymal luminal fluid. As anticipated, significant increases were observed in metabolites such as carnitine and prostaglandins (**Figure 5**). Based on these findings, it is hypothesized that the alterations in the metabolites of the epididymal luminal fluid may be another crucial factor contributing to changes in the expression of sperm flagellar proteins.

Phospholipid metabolites have been identified as inflammatory factors. Arachidonic acid (AA), a major product of phospholipid hydrolysis, plays a significant role in various pathophysiological processes. Furthermore, AA can be converted into various prostaglandin species, with PGD2, along with prostaglandins PGE2, PGI2, and 15-d PGJ2, reported to regulate the inflammation process (21–23). The synthesis of PGD2 in the genitalia is controlled by the enzyme lipocalin-type prostaglandin D2 synthase (L-PGDS) (24), which is highly expressed in the testis and epididymis of mice (25, 26). Reduced expression of L-PGDS has been found in the seminal plasma of oligozoospermic men compared to normozoospermic men (27), indicating its key role in sperm development and maturation. Additionally, PGJ2 has been reported as a metabolite of PGD2 (28), and the metabolites of PGD2 can influence diverse cellular functions.

The absence of tryptophan catabolism along the kynurenine pathway has been shown to produce an inflammatory state in the epididymis (29). Moreover, a significant increase in sperm number and the proportion of sperm with abnormal morphology have been observed in mice deficient in indoleamine 2,3-dioxygenase (IDO), the rate-limiting enzyme of tryptophan catabolism through the kynurenine pathway (29). Kynurenine, a tryptophan-derived metabolite, has reduced concentration in the cauda epididymis fluid of *Cabs1* KO mice compared to WT mice. It is reported that CABS1 is also present in salivary glands and is associated with stress and anti-inflammation (19, 20). The altered metabolomic fingerprinting of inflammatory factors demonstrates an inflammatory state in the epididymis of *Cabs1*-deleted mice.

Lipids are the vital components of sperm, containing cholesterol, phospholipids, and glycolipids. The maturation of sperm involves changes in the lipid composition of the sperm membrane. As spermatozoa move from the caput to the cauda region, there is an increase in membrane fluidity due to a remodeled ratio of cholesterol to phospholipid (30, 31). The increased membrane fluidity in cauda spermatozoa is crucial for sperm movement, acrosome reaction, and egg fusion (31). Additionally, the ratio of polyunsaturated: saturated fatty acids (PUFAs) can contribute to changes in membrane fluidity (30). PUFAs can also alter the composition and organization of mitochondrial membranes, leading to increased production of reactive oxygen species (ROS) and subsequent peroxidation of membrane phospholipids and mitochondrial dysfunction (32). In our study, metabolomic fingerprinting analysis revealed significant decreases in suberic acid, azelaic acid, tetradecanedioic acid, hexadecanedioic acid, and 13-HPODE in KO mice, all of which are lipid or oleochemical products. Disorders of oxidative phosphorylation have been associated with oxidized linoleic acid (33). 13-HPODE, as one of the most common dietary peroxidized lipids, induces inflammation *in vivo* (34) and may be involved in mitochondrial dysfunction-related disorders (35). Abnormalities in lipid metabolism can lead to spermatogenic dysfunction and consequently male infertility.

Furthermore, our study identified high levels of L-carnitine in CABS1-deficient mice. Free L-carnitine is known to be taken from blood plasma and concentrated in the epididymal lumen. The uptake of L-carnitine in the sperm plasma membrane plays a protective role in mitochondrial function, participating in the intermediary metabolism of fatty acids and β-oxidation of long-chain fatty acids (36, 37). Acylcarnitines, fatty acid metabolites, are used as markers for errors in fatty acid oxidation and energy metabolism. Short-chain acylcarnitines (C2-C5) are the most abundant group in the body (38), and altered concentrations have been linked to various diseases and pathologies (39–41). As short-chain acylcarnitines, acetyl-L-carnitine and propionylcarnitine play vital roles in energy production by providing acetyl groups for β-oxidation (42). Carnitine is considered to positively affect sperm motility and maturation by supplying abundance energy and acting as an antioxidant to decrease ROS in mitochondria (43). However, excessive acylcarnitine may reflect inadequate fatty acid oxidation and mitochondrial dysfunction, potentially leading to metabolic disease (44). The increased level of L-carnitine, acetyl-L-carnitine and propionylcarnitine may indicate perturbations in the β-oxidation of fatty acids (45). The quantification of ATP in sperm further supports the notion that disordered energy metabolism may partially account for the decreased sperm motility.

## 5 Conclusion

In our research, we investigated the differences in metabolite profiles of epididymal luminal fluid in male mice with and without CABS1. The analysis of metabolome profiles, combined with ATP quantification, suggested that mitochondrial dysfunction and inflammation may be key factors contributing to the impaired sperm function in *Cabs1* knock-out mice. Further research is required to elucidate the specific role of these metabolites in sperm maturation. This study established a connection between infertility and the metabolite profiles of epididymal luminal fluid, providing insight into the molecular mechanisms of male infertility.

## Data Availability

The raw data supporting the conclusions of this article will be made available by the authors, without undue reservation.
